# Bitter Is Better: Wild Greens Used in the Blue Zone of Ikaria, Greece

**DOI:** 10.3390/nu15143242

**Published:** 2023-07-21

**Authors:** Andrea Pieroni, Gabriella Morini, Maria Piochi, Naji Sulaiman, Raivo Kalle, Shiekh Marifatul Haq, Andrea Devecchi, Cinzia Franceschini, Dauro M. Zocchi, Riccardo Migliavada, Julia Prakofjewa, Matteo Sartori, Nikos Krigas, Mushtaq Ahmad, Luisa Torri, Renata Sõukand

**Affiliations:** 1 University of Gastronomic Sciences, Piazza Vittorio Emanuele II 9, 12042 Pollenzo, Italy; 2 Department of Medical Analysis, Tishk International University, Erbil 44001, Iraq; 3 Department of Ethnology, Charles University, 116 38 Prague, Czech Republic; 4 Estonian Literary Museum, Vanemuise 42, 51003 Tartu, Estonia; 5 Department of Ethnobotany, Institute of Botany, Ilia State University, 0162 Tbilisi, Georgia; 6 Department of Environmental Sciences, Informatics and Statistics, Ca’ Foscari University of Venice, Via Torino 155, 30172 Venezia, Italy; 7 Institute of Plant Breeding and Genetic Resources, Hellenic Agricultural Organization Demeter, 57001 Thessaloniki, Greece; 8 Department of Plant Sciences, Quaid-i-Azam University, Islamabad 45320, Pakistan

**Keywords:** *Chorta*, ethnobiology, food culture, Ikaria, Mediterranean diet, taste perception, extra-oral taste receptors

## Abstract

The current study reports an ethnobotanical field investigation of traditionally gathered and consumed wild greens (*Chorta*) in one of the five so-called Blue Zones in the world: Ikaria Isle, Greece. Through 31 semi-structured interviews, a total of 56 wild green plants were documented along with their culinary uses, linguistic labels, and locally perceived tastes. Most of the gathered greens were described as bitter and associated with members of Asteraceae and Brassicaceae botanical families (31%), while among the top-quoted wild greens, species belonging to these two plant families accounted for 50% of the wild vegetables, which were consumed mostly cooked. Cross-cultural comparison with foraging in other areas of the central-eastern Mediterranean and the Near East demonstrated a remarkable overlapping of Ikarian greens with Cretan and Sicilian, as well as in the prevalence of bitter-tasting botanical genera. Important differences with other wild greens-related food heritage were found, most notably with the Armenian and Kurdish ones, which do not commonly feature many bitter greens. The proven role of extra-oral bitter taste receptors in the modulation of gastric emptying, glucose absorption and crosstalk with microbiota opens new ways of looking at these differences, in particular with regard to possible health implications. The present study is also an important attempt to preserve and document the bio-cultural gastronomic heritage of *Chorta* as a quintessential part of the Mediterranean diet. The study recommends that nutritionists, food scientists, and historians, as well as policymakers and practitioners, pay the required attention to traditional rural dietary systems as models of sustainable health.

## 1. Introduction

The Mediterranean diet (MD)—a nutritional model derived from the cross-cultural epidemiological seven countries study by the American nutritionist Ancel Benjamin Keys [1]—has been attributed to the “food patterns typical of Greece and southern Italy in the early 1960s, where adult life expectancy was among the highest in the world and rates of coronary heart disease, certain cancers, and other diet-related chronic diseases were among the lowest” [2,3,4,5,6]. The MD was recognized as an intangible cultural heritage of humanity by UNESCO a decade ago [7,8]. This food system has been the subject of extensive biomedical literature with important outcomes for public health. The main health benefits of the MD can be resumed in some major effects known to be involved in the origin of non-communicable diseases and thus have an impact on well-being and longevity: (1) lipid-lowering effect; (2) protection against oxidative stress and inflammation; (3) modification of nutrient absorption and metabolism; and (4) modification of microbiota composition [3,4,5,6]. A rough description of ingredients and food products characterizing the MD could be “abundant plant foods, fresh fruit as the typical daily dessert, olive oil as the principal source of fat, dairy products (principally cheese and yogurt), and fish and poultry consumed in low to moderate amounts, zero to four eggs consumed weekly, red meat consumed in low amounts, and wine consumed in low to moderate amounts, normally with the meal” [3,9].

However, the MD is not just a mere list of ingredients or nutritional indications, but a holistic life approach. Since 2010, the pyramid model has also been based on concepts such as seasonality, biodiversity, eco-friendliness, and traditional and local food products [10,11]. The incorporation of these features into the pyramid model represented an element of innovation. Furthermore, food habits are an expression of the existing socio-ecological circumstances associated with a particular place or region [11]. For this reason, it would be more correct to talk about plural MDs. Nowadays, despite the many environmental, human, economic, and social documented benefits, adherence to the MD in Mediterranean basin countries is less than optimal [12]. According to D’Innocenzo et al. [13], it would be crucial to promote awareness of inexpensive local foods, such as traditional wild vegetables, to which people could be oriented in order to appreciate and recognize them without sacrificing taste and pleasure. 

To date, there are five recognized Blue Zones (BZs) in the world, areas where people live healthy, long, and good quality lives: Okinawa (Japan), the Ogliastra area of eastern Sardinia (Italy), the Nicoya Peninsula (Costa Rica), Ikaria (Greece), and Loma Linda (California). 

Dan Buettner and his team detected nine common features of BZs, dubbed the “Power 9” [14]: move naturally (regular physical activity without the need for “heavy workouts”; purpose (the value of knowing your missions and goals); downshift (to reduce chronic stress); 80% rule (stop eating when your stomach is 80% full, in line with frugality); plant slant (prefer plant-based protein, such as legumes, to meat and animal-derived products); wine (moderate consumption of wine, mainly with food and other people); belong (belonging to some faith-based community); loved ones first (caring of the grandparents, parents, life partner, and children); right tribe (relevance of supporting groups, since a “healthy” environment could promote healthy behaviors). 

Comparing dietary models of all five BZs is quite difficult for several reasons, including ethnic and cultural characteristics and lifestyles [15]. A recent review investigated the complexity of the BZ lifestyle, diet in particular, and concluded that it is not possible to promote a specific BZ diet outside the context in which it was born [16]. 

The wild vegetable portion of the traditional Mediterranean culinary system has been described as a “hidden MD” [17] since archaeological studies have been unable to provide robust data on the historical use of wild leafy vegetables (charred plant remains, primarily not including small leaves and leaf rosettes), and these ingredients have been largely ignored by traditional MD bio-nutritional studies. On the other hand, during the past two decades, a considerable number of ethnobotanical field studies in the central and eastern Mediterranean region were centered on the wild plant portion of the MD [17,18,19,20,21,22,23,24,25,26,27,28,29,30]. All of them aimed to document taxonomical identity, vernacular names, plant uses, and, to a much lesser extent, their perceived taste and detailed culinary transformations [31,32,33,34,35,36]. It is well accepted that the widespread use of weedy plants in the MD originated in settlements during the Neolithic Age and that they were used both as food and medicine [37,38]. Details on sensory characteristics of this food heritage and their likely underlying historical development remain largely unexplored. In some of the aforementioned works, it has been qualitatively reported that bitterness intensity was a sort of indicator to distinguish plants used as food from those used as medicine, where medium-bitter taxa were considered both food and medicine [32]. This traditional knowledge found an explanation in the discovery that bitter taste receptors (T2Rs) are also present extra-orally, including in the gastrointestinal tract [39]. Here, bitter receptors can be activated by bitter compounds occurring in food (and are therefore perceived as bitter in the mouth), as well as by endogenous compounds produced during digestion or by microbiota. Interestingly, their activation in extra-oral locations causes systemic responses, like the release of hormones with effects on satiety and glucose control, as well as compounds able to regulate the immune response and the crosstalk with hosted microorganisms, contributing in this way to microbiota composition [40]. Therefore, it would be very interesting to study the contribution of wild plants to the health benefits of the MD at a molecular level, and bitterness seems to be an ideal probe to pursue this aim. 

Many of the MD staple foods are characterized by bitterness or pungency, or both, and studies at the molecular level have established a link between compounds with these sensory properties and some of their beneficial effects on health [41,42,43]. Olive oil, for example, which stands out among other foods because its use was historically confined to the Mediterranean region, contains several phenolic compounds responsible not only for its bitterness [41] and pungency [42] but also for many health effects [43]. Oleocanthal, one of the more representative biophenols of olive oil, which is bitter and elicits a peculiar pungency sensed almost exclusively in the throat [42], has been proven to have anti-inflammatory properties [44]. On the other hand, the rather peculiar sensory properties of these compounds may be ‘aversive’ for certain consumers, to the point that a sort of learning process is required for an individual to develop an appreciation for it [45,46]. Little is currently known about the inter-individual differences in perception of non-cultivated food plants, both in terms of how they are described at a sensory level, as well as why they are liked/consumed by humans. Differences in bitter taste sensitivity across individuals may affect the liking of food, appetite, and, therefore, diet composition, with an impact on nutrient intake [47].

Greece has been a kind of black spot in the field of wild food ethnobotany for many years, even though a large part of the country is considered the home of MD studies [2,3]. To our knowledge, only a few economic botanical and ethnobotanical works focusing on Greek wild greens (*Chorta*) have been published in the international literature [48,49,50,51]. Therefore, we designed and conducted a wild food ethnobotanical investigation on *Chorta* in Ikaria, whose food heritage has been increasingly popularized in the past decade [52]. Systematic and statistically significant cross-geographical comparisons of foraged plant ingredients of the MD are also still lacking, although this could be crucial for a better understanding of the evolution of traditional Mediterranean cuisines and their potential impact on taste perceptions and health. Furthermore, these types of studies could foster the articulation of appropriate food educational strategies and valorization initiatives. 

This study aims (a) to record vernacular plant names and traditional uses of wild vegetables on Ikaria; (b) to compare the Ikarian data with the surrounding countries in those eastern and central Mediterranean regions where ethnobotanical studies on wild vegetables have been conducted in the past few decades; and (c) to propose a conceptual framework for the revitalization of these wild food ingredients. 

## 2. Materials and Methods

### 2.1. Study Area 

**Figure 1:**
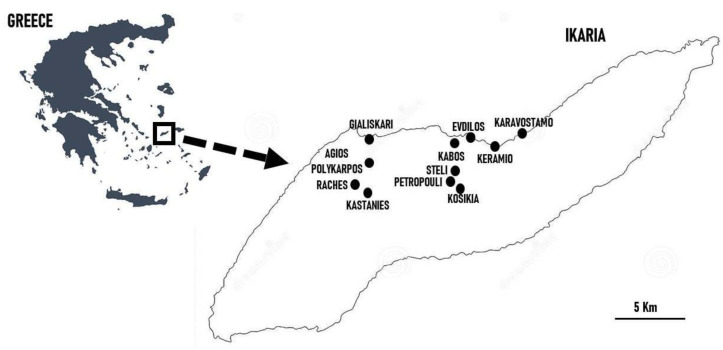
Location of the study area and visited villages on Ikaria Island, Greece.

The field study was conducted in 11 villages across central Ikaria (Figure 1). 

Ikaria is part of the Dodecanese archipelago, in the eastern Aegean Sea. The island has an extremely mountainous topography with the Pramnos–Atheras mountain range being the highest on the island (1048 m.a.s.l.), with green slopes juxtaposing rugged rocks, high mountain streams, small waterfalls, pine and ancient oak forests, and gorges. Its climate is typically Mediterranean, with mild, rainy winters and warm, sunny summers. The average temperature ranges from 10 °C in January and February to 25 °C in July and August. 

**Figure 2:**
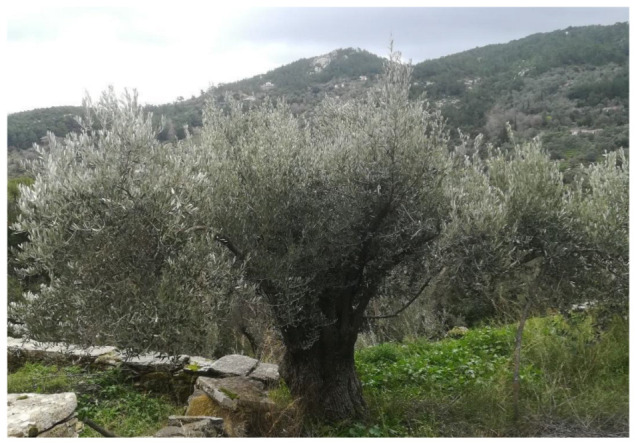
Typical olive orchard in central Ikaria with old trees dominating the agricultural landscape (Photo: A. Pieroni).

The rural Ikarian landscape is characterized by typical olive orchards (Figure 2) and vineyards at lower altitudes, and shrub lands and pastures where sheep and especially goats are herded or, most often, kept semi-domesticated at higher altitudes. Ikaria is home to at least 829 plant taxa, 15 of which are endemic [53,54,55,56]. Nowadays, the local economy is based on small-scale farming, tourism, and the remittances of commuting migrants, who live in Athens most of the year, only returning to Ikaria in the summer to run small tourist businesses.

Ikaria has been inhabited since at least 7000 BC when it was populated by the Neolithic Pelasgians (pre-Hellenic populations). Around 750 BC, Greeks from Miletus colonized Ikaria, establishing a settlement in the area of present-day Campos, which later became the ancient capital city of Oenoe. Despite the sparse population (around 8000), societal integrity has been a distinctive tract of the isle, and Ikarian *Panegyris* (traditional festivals featuring dance, music, and the consumption of local products), teamwork, and elder councils have possibly been the most resilient ones in all of Greece [16]. While poverty and migration have been a feature of the isle until the recent past, the quality of life improved greatly after 1960, when the Greek government began to invest in infrastructure to assist in the promotion of tourism, which today, however, remains much more limited than in most other Greek isles [16].

### 2.2. Ethnobotanical Field Study

The ethnobotanical fieldwork was conducted in January 2023 in the villages shown in Figure 1. The main purpose of the survey was to document local knowledge of wild leafy vegetables (*Chorta*) that are currently collected and consumed by local people. 

**Figure 3:**
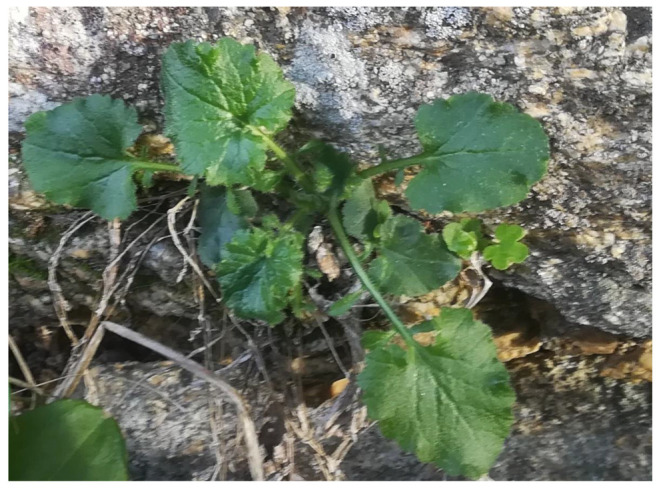
*Lapsana communis* (Photo: A. Pieroni).

Thirty-one study participants were interviewed through a combination of purposive sampling and snowball technique to participate in semi-structured interviews. We mainly targeted middle-aged and elderly people (range: 40 to 84 years), especially farmers, shepherds, and elderly women, since they are considered potential holders of local knowledge in the area. The number of study participants was fairly equal among all the eleven selected settlements. Prior to each interview, verbal informed consent was obtained from each study participant, and the Code of Ethics of the International Society of Ethnobiology [57] was rigorously followed. Semi-structured interviews were conducted in the local language (Greek) or English. We documented local name(s) and detailed food uses for each of the free-listed wild greens. We intentionally excluded mushrooms as well as wild fruits and snacks (i.e., wild plants mainly consumed for leisure outside food contexts or while walking in the wild) from the survey. The quoted wild food taxa were collected from the study area, when available, and identified by R. Kalle and N. Krigas using standard reference works on the Aegean flora [53,54,55,56]. Plant specimens (Figure 3) were collected whenever possible and voucher specimens (with numbers UVVETBOTIK01-38) were deposited at the Herbarium of the Bio-Cultural Diversity Lab of the Department of Environmental Sciences, Informatics and Statistics, Ca’ Foscari University of Venice, Italy. The identification of wild taxa, which were not available during the fieldwork, was conducted based on folk names and detailed descriptions of plant species (those botanical taxa do not bear voucher codes); in such cases, we showed photographs of the presumed plants to the study participants after a preliminary evaluation of the quoted local name and its description. Moreover, a comparison of local phytonyms was carried out with the records collected in our previous study conducted in Crete, in which we linked them to botanical vouchers and identities [58]. The World Flora Online database was followed for the nomenclature [59], while plant family names were consistent with the Angiosperm Phylogeny Website [60]. Recorded local Greek names were transcribed in the Latin alphabet directly from the phonetic transcriptions of the original recordings.

### 2.3. Cross-Cultural Data Analysis

**Figure 4:**
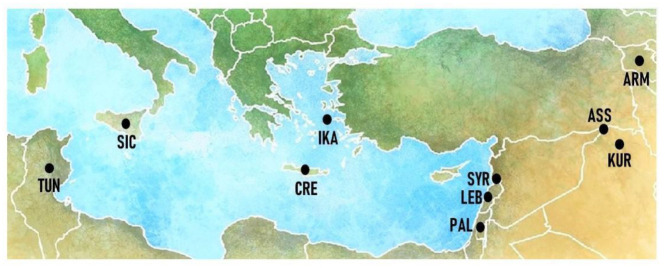
Central-eastern Mediterranean and Near Eastern field study areas used in the comparative analysis; letter codes refer to the considered sites reported in Table 1.

A cross-cultural comparison was conducted by analyzing the data collected in the present study together with those reported by previous ethnobotanical studies on wild foods during the past decade in central/eastern Mediterranean and Near Eastern regions: coastal Syria (SYR), Lebanon (LEB), Palestine (PAL), Assyria (ASS), Kurdistan (KUR), Armenia (ARM), Crete (CRE), Sicily (SIC), and Tunisia (TUN) (Figure 4). We only considered field studies in which quotation indexes or at least clear indications about the most popular wild greens were reported. All considered studies focused on small communities (number of interviews between 20 and 50). In contrast, two studies (in Lebanon and Sicily) referred to large-scale surveys (151–980 informants) conducted over many years (2018–2022 and 2005–2015, respectively) [29,61]. Despite these discrepancies, we decided to include them because other suitable ethnobotanical investigations with quotation indexes were lacking in these two areas. Lastly, the results were also discussed with those reported in recent years among Assyrians in Iraqi Kurdistan and along the Syrian-Turkish border [62,63], since most researchers tend to agree with the *theory of continuity* between modern Assyrian people and ancient Assyrian people, i.e., Mesopotamian Neolithic farmers [64]. 

We deliberately avoided considering ethnobotanical works based on market surveys only, as they cannot reveal the actual domestic plant uses and their cultural salience within households, simple reviews on wild food plants, and ethnobotanical studies without clear evidence of genuine ethnographic and in-depth ethnobiological methods [65]. Moreover, in this analysis, we considered botanical genera and not botanical species or subspecies, as we wanted to mitigate the effect of possible differences in ecological occurrences of single species within the same genera in different areas.

### 2.4. Statistical Analysis

For the cross-cultural comparison, a matrix was built in which the top quoted wild greens (WGs) genera were reported in rows, while the local use/non-use, as well as the sensory classification, were reported in columns. From a sensory point of view, plants were classified as ‘bitter/pungent’, ‘non-bitter’, or ‘aromatic’ (respectively: ‘pik’, ‘gly’, and ‘myr’ as shown in Table 2). In Ikaria and Crete, this classification was emic, i.e., based on interviews with respondents, depending on how they spontaneously described WGs, while for the other locations, the sensory classification was assigned on the basis of the botanical identity (etic perception). 

We used Pearson’s measure of correlation to calculate the correlation between the 10 study sites based on species use and non-use, as well as the sensory characterization. The Pearson correlation coefficient (rho), whose value ranges from −1 (perfect negative correlation) to +1 (perfect positive correlation), with 0 indicating no linear correlation, evaluates the degree and direction of the association between the study locations (see Figure 4). We performed the Pearson correlation analysis in Origin Pro software version 10. For comparing the relationships between the study sites and the use/non-use of WGs genera, we employed a detrended correspondence analysis (DCA), which is an ordination technique supported by reciprocal averaging. DCA is an eigenvector ordination technique used to detect the length of the gradient and the relationship among biological types [70]. In DCA, data are transformed to remove the linear trend, allowing the analysis to focus on the nonlinear relationships between the variables. This can be useful in identifying underlying patterns in complex data sets; DCA was performed in CANOCO version 4.5.

Multiple correspondence analysis (MCA) is a multivariate statistical method aimed at detecting groups of individuals with similar profiles, as well as the associations between several categorical variables. MCA generalizes simple correspondence analysis, which is limited to two categorical variables [71]. It also generalizes principal component analysis when the variables are categorical and not quantitative [72]. We applied MCA to the data in Table 3 to investigate the association among the sensory classification (ar, b, and nb) and considered areas (IKA, CRE, LEB, SYR, TUN, SIC, ARM, PAL, ASS, and KUR). We also used MCA to investigate the similarities/dissimilarities among units (wild greens). From a statistical perspective, the variable “sensory classification” is categorical, and the variable “areas” may be represented as several, and therefore as dichotomous, categorical variables (presence/absence of local food uses). We carried out the MCA in R software using the function MCA in the FactoMineR package [73,74]. For data visualization, we used the Factoextra package [75]. 

The biplot is the graphical output of MCA. The distance between any pair of row or column points provides a measure of their similarity (or dissimilarity). Row points with similar profiles are close to each other on the factor map. The same holds true for column points. The first two dimensions retain 46.5% of the total inertia (variation) contained in the data. The quality of the representation is called the squared cosine (cos^2^) and measures the degree of the association between variable categories and a particular axis. If a variable category is well represented by two dimensions, the sum of the corresponding cos^2^ is close to one. 

## 3. Results and Discussion

### 3.1. Ikarian Chorta

The wild greens-based food heritage of central Ikaria includes fifty-six botanical taxa, of which fifty-five were identified (Table 2). 

Most of the reported species were bitter Asteraceae (members of 14 genera) and pungent/bitter Brassicaceae (3 genera). Among other bitter compounds, edible Asteraceae contain sesquiterpenoids, which are considered to be chemosystematic markers of them [76,77], while Brassicaceae contain glucosinolates (bitter) and precursors of isothiocyanates (pungent), generally making these plants both bitter and pungent [78]. 

This picture is similar to the one reported one century ago by von Heldreich [48] in the first economic botanical survey conducted in Greece and in a more recent investigation we carried out in Crete [58], as well as in the other wild food ethnobotanical studies of the Mediterranean [17,18,19,20,21,22,23,24,25,26,27,28,29,30]. 

**Figure 5:**
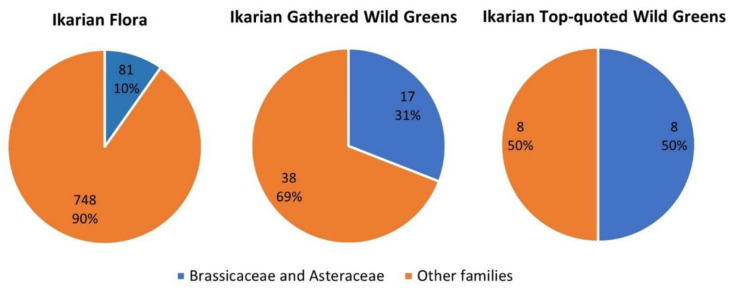
Bitter/pungent Asteraceae and Brassicaceae species in the Ikarian flora and among the traditionally gathered Ikarian wild greens.

In Figure 5, we report the proportion of bitter/pungent Asteraceae and Brassicaceae in the overall Ikarian flora among the traditionally gathered Ikarian wild greens and the top quoted Ikarian wild greens.

The disproportion between the ecological and cultural dimensions of these two botanical families is very clear. In fact, despite Asteraceae and Brassicaceae not being highly represented in the Ikarian flora, these families were among the most quoted. This shows that Ikarian inhabitants look predominately for bitter/pungent wild greens.

**Figure 6:**
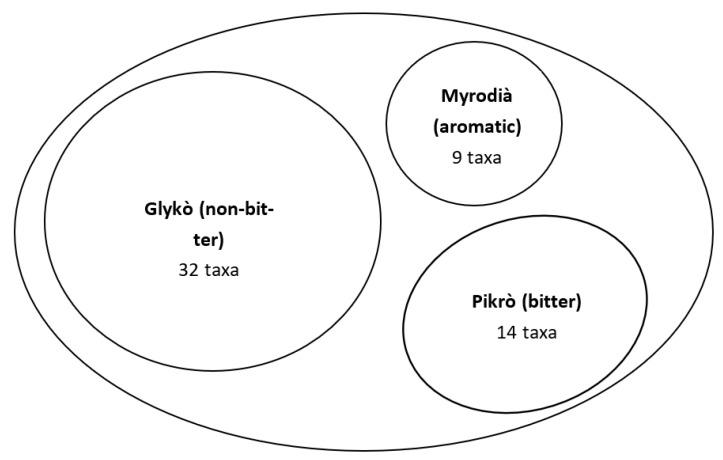
Folk classification of wild greens (*Chorta*) according to their sensory classification as perceived among Ikarian people.

Bitter taste seems to have played an additional crucial role in shaping the cognition of wild greens. In the study area, all our participants described wild greens as *Chorta*; however, the cognitive categories underpinned in the folk classification of *Chorta* are linked to the perceived taste. Respondents showed remarkably high agreement in classifying plants as ‘bitter/pungent’, ‘non-bitter’, or ‘aromatic’ (Figure 6). The term ‘bitter’ technically refers to one of the so-called basic tastes [79], the detection of which is due to at least 25 receptors (members of the G-protein-coupled receptors superfamily) expressed in taste receptor cells in the oral cavity [80]. In contrast, the term ‘pungent’ refers to a chemesthetic sensation, precisely pungency, due to the activation of transient receptor potential ankyrin 1 (TRPA1), one of the about 30 TRPs identified in mammals [81]. Pungency is often also described as burning or oral irritation. The term ‘aromatic’ may refer to the volatile fraction, which is responsible for the sensation of smell through the activation of olfactory receptors in the olfactory bulb both ortho-nasally (e.g., when smelling food) and retro-nasally (e.g., when swallowing food) [82,83,84].

**Figure 7:**
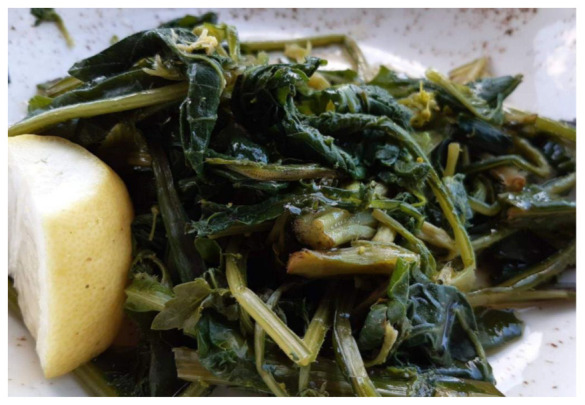
Typical culinary preparation of local *Chorta* on Ikaria Island (Photo: A. Pieroni).

In Ikaria, most wild greens are consumed boiled (then seasoned with olive oil and lemon) (Figure 7) and in pies; preparations with eggs are also popular.

### 3.2. Cross-Cultural Comparisons

The cross-cultural comparison of the collected Ikarian data with those from other Mediterranean and Near Eastern sites was conducted by building a matrix (Table 3) in which only botanical genera that were recorded as very commonly quoted or used in the study sites were considered. 

**Figure 8:**
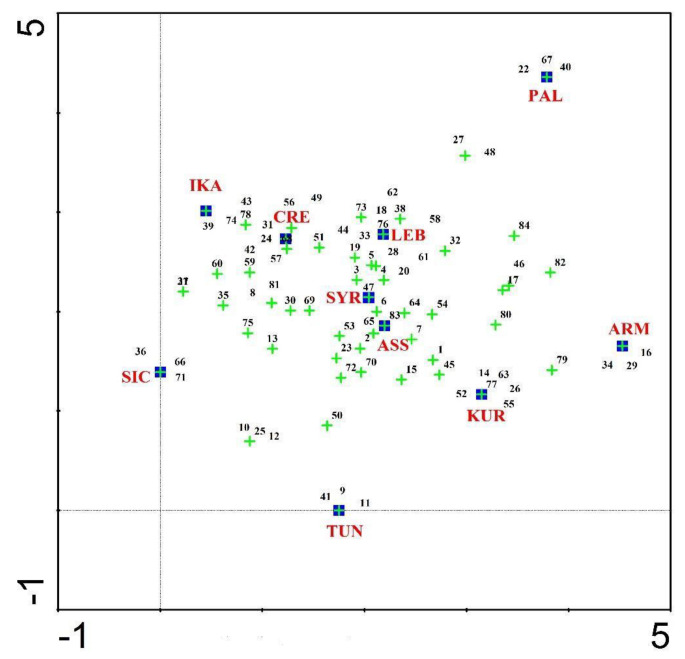
Detrended correspondence analysis (DCA) concerning the comparison between the gathered and consumed Ikarian wild greens (plant genera) and those of the other considered sites (the letter codes correspond to those in Table 1 and Figure 4).

**Figure 9:**
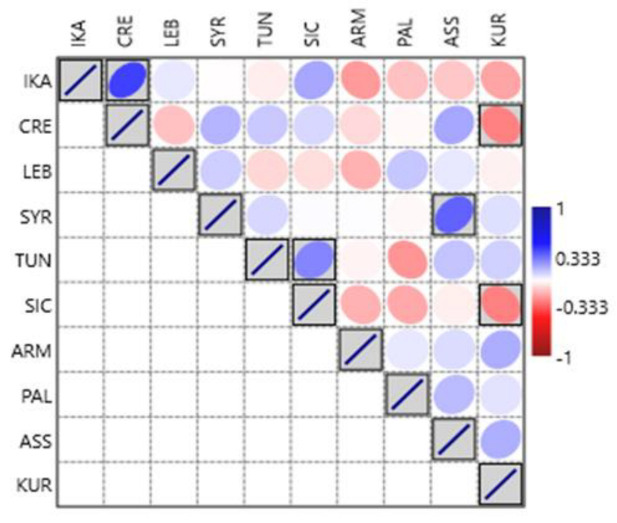
Similarities of the Ikarian gathered and consumed wild greens with those of the other considered sites (the letter codes correspond to those in Table 1 and Figure 4).

Comparisons between the collected Ikarian data with those from other Mediterranean and Near Eastern sites (referring to the use/non-use of the top quoted wild greens) are shown in Figure 8 and Figure 9. Ikarian wild greens show the highest similarity with the Cretan ones and, to a lesser extent, the Sicilian ones. Some similarities with the Syrian, Assyrian, and Lebanese data also emerged, while the highest dissimilarities were found between the Ikarian data and those from Armenia, Kurdistan, Palestine, and Tunisia. 

### 3.3. Bitter Is Better

**Figure 10:**
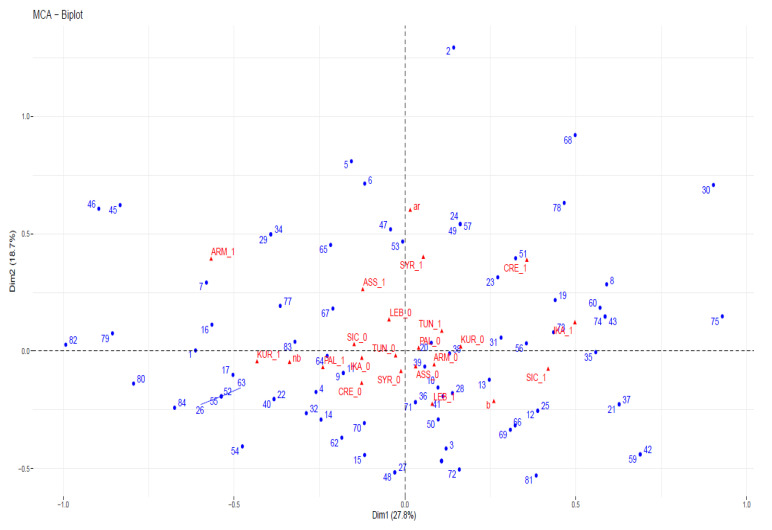
Biplot representation of the multiple analysis based on the matrix in Table 3; 1 refers to the existence of a local food use, 0 to its absence (empty cells in Table 3).

**Figure 11:**
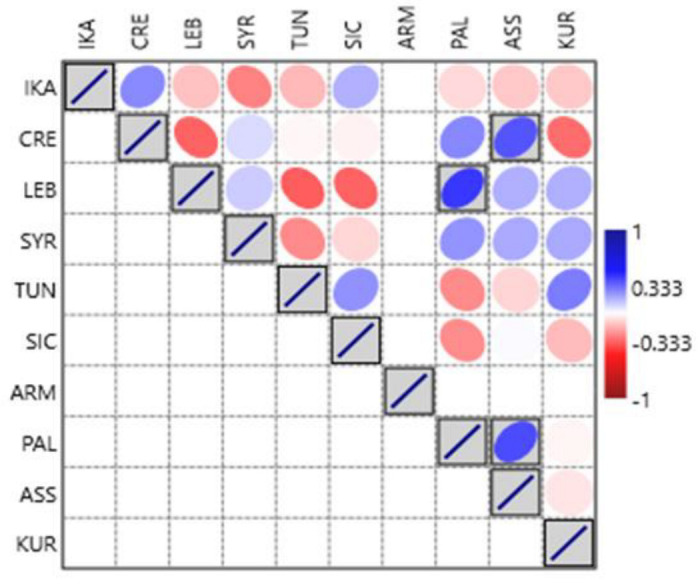
Similarities of the wild greens in the considered sites (based on the matrix in Table 3) based on their tastes.

When the comparative analysis was conducted considering the sensory classification, the overall picture described in the previous paragraph became clearer and somewhat surprising (Figure 10 and Figure 11). In the plot, we limit ourselves to mentioning that the sums of virtually all cos^2^ are greater than 0.80, and the best representations in two dimensions are given by the variables ar, b, and CRE; the corresponding cos^2^ are always greater than 0.70. Variable categories that contribute the most to the first two dimensions constitute the best summaries of the variability in the data set. They are ar (26.6%), CRE_1 (23.95%), ARM_1 (18.83%), b (16.11%), IKA_1 (14.27%), KUR_1 (13.67%), SIC_1 (12.65%), and SYR_1 (11.21%).

The comparison of the reported sensory description of Ikarian wild greens again shows major similarities to those of Sicily and Crete. There are a few considerations that appear from the data that are particularly relevant and complex: one concerning Armenians and another concerning Assyrians. The remarkable absence of top-quoted bitter wild greens among Armenians is interesting and can only be partially linked to the fact that Armenians have a higher proportion of PROP *tasters* [85] than other neighboring populations. So-called *tasters* are individuals that perceive the bitterness of the non-natural compounds PTC (phenylthiocarbamide) and PROP (6-n-propylthiouracil), both of which activate the bitter taste receptor T2R38, as highly intense. For this bitter taste receptor, two main alleles in populations worldwide are known [86]: the PTC *taster* PAV (encoding proline, alanine, and valine at the respective variant sites) and the PTC *non-taster* AVI (encoding alanine, valine, and isoleucine at these sites). T2R38 is also one of the bitter receptors activated by most glucosinolates and some isothiocyanates [78]. 

Certain studies reported that the bitterness of PROP positively correlates with the bitterness intensity of glucosinolates in broccoli juice [87] and that specific genotypes for gene T2R38 (PAV/PAV) are associated with an increased bitterness of glucosinolates-containing foods [88]. However, other studies did not find a relationship between PROP sensitivity and the intake of cruciferous vegetables when considering ethnically diverse children [89]. In fact, the perception of oral bitterness is complex, and the more than 25 receptors for bitter molecules in food have a complex tuning [90]. Moreover, plants belonging to different families (e.g., Asteraceae and Brassicaceae) contain different secondary metabolites activating different bitter receptors, and covariation in responsiveness to these compounds has been poorly documented so far, especially from a cross-cultural perspective.

The data found in the current research warrant further investigation in additional studies and in different directions. Even if it is true that the perception of bitterness (also including its genetic factors) has a role in food preferences, we know that learning plays a major part in what is identified as food, as we learn to like certain foods. In addition, humans learned that bitterness is a hallmark of bioactivity: we used this sensory property as a means to select non-cultivated plants with positive effects on health, with this benefit outweighing their initial unpleasantness. A similar process has been suggested as the driving force for positively selecting food characterized by hotness and pungency [91,92]. 

With regard to the MD, the fact that a “good” olive oil should be bitter and pungent represents an adequate tasty example: such attributes have long been traditionally used as quality markers, but this has only recently been demonstrated at a molecular level [93]. Moreover, recent studies have demonstrated that bitter and pungent phytochemicals have a higher probability of exerting anti-inflammatory effects than other secondary metabolites [94]. 

One of the directions to be explored to find a possible explanation for these differences lies with exercised culinary practices, as cuisine is not only a matter of ingredients, but ultimately how these ingredients are combined and treated. Moreover, such practices usually play a significant role in making the final preparation not only edible (consumable) for people (with their genetics) living in a certain place, but also pleasant for them (with their genetics and culture). Therefore, we have to look at local cuisines as bio-cultural processes able to incorporate experiences into procedures, resulting from a long sequence of cultural assimilation following body assimilation, with an observation of health effects being performed, not only in the short term (trial and error, acute toxicity) but also in the long term (well-being and health) [95]. In this sense, traditional cuisines and the concomitant diets they generate are the best longitudinal studies to which we can refer. 

An example of the role that different culinary practices could have in the sensory acceptability of a wild plant comes from a study demonstrating how the perception of the bitter sesquiterpene lactones compounds in Asteraceae is different at different pH levels: it is enough to add lemon to a plant of this family to mitigate the bitterness of these compounds (also proven by in vitro tests) [76], making it more acceptable. However, in the intestine, the pH is different (less acidic), and, therefore, the activation of bitter receptors expressed there will be possible, with all the associated health benefits. It is worth noting that in Ikaria most of the boiled *Chorta,* including bitter ones, are consumed with lemon, and this is consistently perceived as a culinary “must” by elderly community members. 

**Figure 12:**
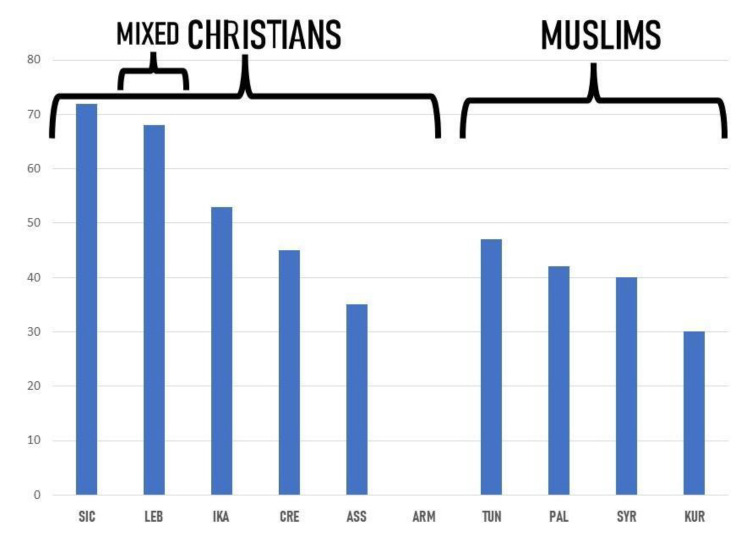
The proportion of the top-quoted bitter/pungent wild greens (botanical genera) among the overall top-quoted wild greens in the considered sites (the letter codes correspond to those as in Table 1 and Figure 4).

Apart from the Armenian data, an interesting link emerges between the Christian faith and the detected major preference for bitter greens (Figure 12). This may suggest that the tradition of fasting during Lent could have enhanced the popularity of wild greens foraging and consumption during the spring, as our study participants pointed out and as qualitative unpublished data gathered among mixed Muslim and Christian coastal Syrian communities have confirmed. It is known that in the Bible the term ‘bitter’ often appears. For example, in Exodus 12:8, Israelite families were called by God ‘*to eat meat roasted over the fire, along with bitter herbs, and bread made without yeast*’. So rather than a savory seasoning for meat, God’s call for bitter greens was meant to serve as a reminder of the bitter existence of Jews under the Pharaoh’s oppressive rule. In this sense, bitterness could be seen as a memory carrier of the sacrifices and in opposition to ostentation, which may have been adopted later by Christians. Other religious factors may have influenced the consumption of specific herbs by some Muslim communities in the Near East. Al-Hakim Bi Amr Allah, one ruler of the Fatimid Caliphate (an empire that ruled the southern and eastern Mediterranean between 909 and 1171 AD), prohibited the eating of *molokhia* (*Corchorus olitorius* L.) for religious symbolic reasons. This ban was subsequently followed by Ismaili and Druze communities for some time. For similar reasons, the consumption of *Nasturtium officinale* was not recommended/preferred [96].

However, the fact that Christian Assyrians (descendants of the first Neolithic settlers) do not seem to particularly like bitter greens is surprising and needs further investigation. The Neolithic diet, which introduced bitter/pungent weeds into human nutrition, could have originated in the Fertile Crescent without necessarily privileging bitter greens at its inception. Instead, bitter wild greens could have become more crucial when Neolithic dietary systems, which moved westward across the Mediterranean basin a few thousand years BC, were influenced by Hebrew food perceptions and, later, by the spread of Christianity and its fasting prescriptions during Lent. In fact, early Christian communities strictly observed fasting as a common religious practice [97], which is believed to have originated from Jewish religious culture [98]. The practice of abstaining from certain foods or drinks, however, was already present in late antiquity [99], although it did not spread consistently [100]. In the modern age, the Catholic Church maintained fasting or tough dietary prescriptions until the 20th Century, such as on Fridays, Ash Wednesday, and during Lent [101]. Throughout the modern era [102], fasting during Lent has been challenged by Protestantism, but it has remained an essential practice in affirming the identity of Mediterranean Christian religious communities [103], especially in eastern Churches [101,104,105,106]. The dietary rules of Greek Orthodox Christianity have, in fact, significantly influenced the Greek Mediterranean diet [107].

### 3.4. How to Make Food Heritage of Wild Greens Trendy Again

The wild plant portion of Mediterranean diets is not only an important part of the local biological and cultural heritage, but also a significant driver of small-scale economies. A few of the recorded plants were available in local farmers’ markets, and in the study sites, they are on the menu of almost every *taverna*, where in the summer many tourists enjoy homemade Greek dishes of the MD. These incipient dynamics of valorization could open up promising opportunities to promote these key elements of the MD at a local level, which, to date, have been poorly considered and not valorized in the framework of institutional projects. Unlike other contexts where attempts to promote MD took place [108], the Ikarian scenario could follow a more bottom-up and participatory path towards the revitalization of this corpus of food and associated sociability. The resilience of the heritage of *Chorta* is remarkable in Ikaria, but the local knowledge and practices linked to these wild greens are mainly in the hands of elderly community members. It is therefore crucial to find tools and frameworks that can facilitate the transmission of this knowledge to newer generations. 

Starting from the baseline data regarding the material and immaterial elements connected to the local foodscape and its gastronomy (i.e., ingredients, products, and dishes, as well as their production, preparation, and consumption methods) and the understanding of the continuous changes in these dietary systems, this process could develop around a strong involvement of local actors and the creation of inclusive platforms that also involve potential newcomers/external agents (e.g., social and cultural entrepreneurs and innovators).

Although most of the study participants agreed that young people no longer know about *Chorta*, we observed some young Ikarian farmers actively working to place sustainable local food, organic farming, and wild greens once again at the center of their lives. This may also be due to the socio-cultural context of Ikaria, which, from being one of the poorest islands of Greece after World War II, has increasingly, during the past two decades, turned into an important hub for many youngsters in Greece and southern Europe, who have wanted to reconnect to nature via social projects and reject mass tourism and capitalist consumerism. The sociability and food-sharing customs linked to *Panegyris* (traditional social gatherings and feasts, nowadays celebrating Saints’ name days and other religious Greek Orthodox Christian holidays) generate the iconic Ikarian cross-generational conviviality. The Ikarian social well-being acquired via this profound culture of commensality is possibly also one of the factors making the Ikarian diet so valuable, as discussed in other works [14,16,109], and a precious heritage itself. In Ikarian society, personal relations have a high value in general. According to Zouras [110], an 89-year-old woman described her relationship with neighbors in this way:

“They say here ‘your neighbor is your brother’ but sometimes your neighbor is more important because there is someone to go to if there’s an emergency…”. 

Zouras [110] outlines five elements contributing to the higher socialization of vegetable gardening and foraging, and the presence of places supporting social interactions in common spaces deserve to be specifically stressed. Those spaces for meeting with the neighbors and other community members could serve for the future promotion of knowledge on wild vegetables, expressed in the form of informative installations and pre-planned meetings held by elders introducing wild greens and their uses to younger community members, followed by joint preparations in traditional and innovative ways. Creating a space for knowledge exchange in order to preserve a practice that is crucial from a health and food security perspective would be an important contribution from elderly community members, potentially motivating younger generations to explore and bring the traditional practice up to date. The survival and revitalization-in-practice of wild greens in Ikaria should pass through various ad hoc initiatives devoted to youngsters, both in terms of environmental and food education. Young people are increasingly conscious of environmental and health-related issues and may be more open to trying new foods if they find them beneficial to their well-being. Providing information about the ecological role and nutritional benefits of wild greens can contribute to increasing their consumption among young people. Local initiatives devoted to younger generations in the Mediterranean region could promote how to gather, clean, cook, and serve wild vegetables, making their inclusion in the daily diet easier. Indeed, it has been shown that cooking skills are positively correlated with vegetable consumption, healthier food choices, and increased adherence to nutritional guidelines [111]. 

To some extent, bitter foods and beverages are slowly re-gaining interest in the gastronomic panorama (i.e., beers characterized by a high level of bitterness, vermouth and related bitter wines and spirits, and chocolates with less sugar). Some well-known chefs are using bitter ingredients (many of which are foraged) to create more complex and unique dishes; therefore, bitterness has been the subject of some articles in magazines, such as the one in a recent issue of the magazine “The New York Times” [112]. Young foodies seem to have started to not dislike bitter food in principle: in a sample of healthy young people recruited among students at the University of Gastronomic Sciences (Bra, Italy), bitter-philic consumers outnumbered bitter-phobic ones (36.4% vs. 24.2%) [113].

## 4. Conclusions

This study recorded the wild greens-centered food and cultural heritage of central Ikaria, Greece. Most of the recorded species were frequently consumed during the spring, either boiled and seasoned with olive oil and lemon or prepared in savory pies. Cross-cultural/regional comparisons suggest that Ikarian *Chorta* customs have strong links to those of Crete and Sicily and may have evolved from an original “bulk” of weedy vegetables used by Neolithic farmers in the Near East, but from which the recorded heritage also shows significant differences. Possible cultural factors (culinary practices and religious prescriptions) underpinned in the observed commonalities and differences between Ikarian and other Mediterranean and Near East wild greens are considered and discussed as well. 

The current research may provide a baseline that could contribute to a better understanding of the links between the wild plant portion of the MD, taste perception, and health in the Mediterranean and the Near East. Therefore, it constitutes the starting point for further studies to possibly identify the phytotastants in non-cultivated plants, the molecular structures with which they interact (receptors), and their variability between individuals in relation to health status. Future nutrition studies are strongly recommended to concern health-promoting properties associated with the consumption of wild green plants. Ultimately, fostering more research and evidence that “bitter is better”, both at the cultural and the molecular level, could inspire and strengthen programs aimed at promoting public awareness about the importance of bitter food heritage for improving health and well-being.

## Figures and Tables

**Table 1:** Details of the previous ethnobotanical field studies on traditional wild greens used for the comparative analysis (see Figure 4).

Site Code	Area and Country	Main Ethnicity of the Considered Sample	Religion of the Considered Sample	Reference
IKA	Central Ikaria, Greece	Greeks	Orthodox Christians	(Present study)
CRE	Central Crete, Greece	Greeks	Orthodox Christians	[58]
SIC	(entire) Sicily, Italy	Italians	Catholic Christians	[29]
TUN	Sidi Bouzid area, Tunisia	Arabs	Sunni Muslims	[66]
SYR	Tartus area, Syria	Arabs	(mainly) Alawite Muslims	[67]
LEB	(entire) Lebanon	Arabs	(Various denominations of) Christians and Muslims	[61]
PAL	Northern West Bank, Palestine	Arabs	(mainly) Sunni Muslims	[68]
ASS	Syrian-Turkish borderland and NW Kurdistan region, Northern Iraq	Assyrians	Eastern Christians	[62,63]
KUR	Kurdistan region, Northern Iraq	Kurds	Sunni Muslims	[62]
ARM	Central Armenia	Armenians	Orthodox Christians	[69]

**Table 2:** Reported Ikarian wild greens and their local names, local culinary uses, reported perceived sensory classification, and frequency of quotation.

Botanical Taxon or Taxa; Botanical Family (Botanical Voucher Specimen Code)	Recorded Local Name(s) (in Singular or Plural)	Used Parts	Local Sensory Classification	Local Culinary Uses	Quotation Index
*Alcea rosea* L.; Malvaceae (IK17)	Molocha	Leaves	Gly	Boiled	R
*Allium ampeloprasum* L.; Amaryllidaceae (IK28)	Agriopraso	Whole plant	Myr	Pies cooked with rice	C
*Allium vineale* L.; Amaryllidaceae	Agrioskordo	Whole plant	Myr	Seasoning	R
*Amaranthus blitum* L. and possibly other *Amaranthus* species; Amaranthaceae	Vlito	Leaves	Gly	Boiled, stewed with tomatoes	VC
*Anchusa hybrida* Ten.; Boraginaceae (IK36)	Shipita	Young aerial parts	Gly	Salads, boiled	C
*Asparagus acutifolius* L.; Asparagaceae (IK27)	Agriasfaragia	Shoots	Pik	Cooked with eggs	C
*Calendula arvensis* L.; Asteraceae (IK01)	Not recorded	Leaves	Gly	Boiled	R
*Carduus pycnocephalus* L.; Asteraceae (IK10)	Gaidoroga, Agathi	Young stems	Pik	Boiled	C
*Crithmum maritimum* L.; Apiaceae (IK29)	Kritamo	Young aerial part	Gly	Pickled, salads	C
*Cichorium spinosum* L.; Asteraceae	Stamnagathi	Rosettes	Pik	Salads, boiled	C
*Cirsium vulgare* (Savi) Ten.; Asteraceae (IK11)	Gaidoraga, Agathi	Young leaves	Pik	Boiled	C
*Colocasia esculenta* (L.) Schott; Araceae *	Glikopatates	Young leaves	Gly	Diverse wrapping material in cooking	R
*Crepis fraasii* Sch.Bip. (IK33), *C. sancta* (L.) Bornm. (IK15), and possibly other *Crepis* spp.; Asteraceae	Radiki	Rosettes	Pik	Boiled	VC
*Cucurbita maxima* Duchesne; Cucurbitaceae *	Kolokithi	Shoots	Gly	Boiled	R
*Cynara cardunculus* subsp. *cardunculus*; Asteraceae	Agriaginara	Flower receptacles	Gly	Salads, boiled	C
*Daucus carota* L.; Apiaceae	Agriokaroto	Young aerial parts	Myr	Pies	C
*Dioscorea communis* (L.) Caddick and Wilkin; Dioscoreaceae	Ovries	Shoots	Pik	Cooked with eggs	C
*Echium plantagineum* L.; Boraginaceae	Voidoglossa	Leaves	Gly	Boiled	R
*Eruca vesicaria* (L.) Cav.; Brassicaceae (IK05)	Roka	Young aerial parts	Pik	Salads	C
*Ficus carica* L.; Moraceae	Agriasikia	Unripe fruits	Gly	Preserves	C
*Foeniculum vulgare* Mill.; Apiaceae (IK22)	Maratho	Young aerial parts	Myr	Boiled and pies, dolmade filling	VC
*Galium rotundifolium* L.; Rubiaceae (IK26)	Kolitzida	aerial parts	Gly	Boiled	R
*Hyoseris scabra* L.; Asteraceae (IK07)	Radiki	Rosettes	Pik	Boiled	VC
*Hypochaeris glabra* L.; Asteraceae (IK32)	Glikosiridi, Radiki	Rosettes	Gly	Boiled	C
*Ipomoea batatas* (L.) Lam.; Convolvulaceae *	Glikopatates	Shoots	Gly	Boiled	R
*Lapsana communis* L.; Asteraceae (IK02)	Petrouchia, Petrukki	Rosettes	Gly	Boiled and pies	C
*Leontodon tuberosus* L.; Asteraceae (IK14, IK25)	Radiki, Pikrosiridia	Rosettes	Pik	Boiled	VC
*Lupinus albus* L.; Fabaceae (IK30)	Loumbina	Shoots	Gly	Salads	R
*Malva neglecta* Wallr. (IK18), and *M. parviflora* (IK37); Malvaceae	Molocha	Leaves	Gly	Boiled	R
*Mentha longifolia* (L.) L.; Lamiaceae	Diosmos, Iosmos	Leaves	Myr	Seasoning	C
*Muscari comosum* (L.) Mill.; Asparagaceae	Vorvos	Bulbs	Pik	Boiled and pickled	VC
	--	Aerial parts	Gly	Boiled	R
*Origanum onites* L.; Lamiaceae (IK31)	Rigani	Flowering tops	Myr	Seasoning	VC
*Papaver rhoeas* L.; Papaveraceae	Paparouna	Rosettes	Gly	Boiled and pies	VC
*Portulaca oleracea* L.; Portulacaceae	Glistrida	Aerial parts	Gly	Salads	VC
*Raphanus raphanistrum* L.; Brassicaceae (IK06)	Vrouves	Young aerial parts	Pik	Boiled and pies	VC
*Reichardia picroides* (L.) Roth; Asteraceae (IK21)	Galatsida	Rosettes	Gly	Boiled and pies	VC
*Rumex conglomeratus* Murray; Polygonaceae (IK20)	Lahanes	Leaves	Gly	Boiled, cooked with eggs, pies, dolmades	C
*Rumex pulcher* L.; Polygonaceae (IK35)	Lapatho	Leaves	Gly	Boiled	R
*Satureja thymbra* L.; Lamiaceae	Thimbra	Aerial parts	Myr	Seasoning	R
*Scolymus hispanicus* L.; Asteraceae	Askolimbros	Young aerial parts	Gly	Boiled	C
*Silene vulgaris* (Moench) Garcke; Caryophyllaceae (IK19)	Strifulia	Young aerial parts	Gly	Pies	C
*Sinapis alba* L.; Brassicaceae (IK13)	Vrouves	Young aerial parts	Pik	Boiled and pies	VC
*Solanum nigrum* L.; Solanaceae (IK09)	Stifno	Leaves	Pik	Boiled	C
	fruits	Fruits	Gly	Chutney	R
*Sonchus oleraceus* L. (IK12), and possibly other *Sonchus* spp.; Asteraceae	Tzochos	Young aerial parts	Gly	Boiled and pies	VC
*Taraxacum minimum* (V.Brig.) N.Terracc. (IK16), and possibly other *Taraxacum* spp.; Asteraceae	Radiki, Pikrosiridia	Rosettes	Pik	Boiled	VC
*Tordylium apulum* L.; Apiaceae	Kaftkalithra	Rosettes	Myr	Salads, boiled	VC
*Scandix pecten-veneris* L.; Apiaceae (IK34)	Kaftkalithra	Rosettes	Myr	Salads, boiled	VC
*Tragopogon porrifolius* L.; Asteraceae	Tragopogon	Young aerial parts	Gly	Pies	R
*Urospermum picroides* (L.) Scop. ex F.W.Schmidt; Asteraceae	Agnosiridio, Glikochorto	Rosettes	Gly	Boiled	C
*Urtica membranacea* Poir. ex Savigny; Urticaceae (IK23)	Tzouknides	Young aerial parts	Gly	Boiled and pies	C
*Vicia faba* L.; Fabaceae *	Kukkia	Shoots	Gly	Boiled	R
*Vitis vinifera* L.; Vitaceae *	Ampeli	Shoots	Gly	Boiled	R
Unidentified taxon	Drosseridia	Rosettes	Gly	Boiled	R

* Cultivated taxa (used in “uncommon” ways); Gly = glykò: non-bitter; Pik = pikrò: bitter; Myr = myrodià: aromatic; VC = very commonly quoted: quoted by 40–100% of the study participants; C = commonly quoted: quoted by 10–39% of the study participants; R = rarely quoted: quoted by less than 10% of the study participants.

**Table 3:** Matrix comparing the top quoted Ikarian wild greens (botanical genera) and their sensory classification with those of the other comparative sites (see Figure 4 and Table 1).

Wild Greens	Sensory Classification	IKA	CRE	LEB	SYR	TUN	SIC	ARM	PAL	ASS	KUR
*Alcea*	nb									1	1
*Allium*	ar		1		1	1	1	1		1	
*Amaranthus*	b	1		1							1
*Anchusa*	nb			1	1					1	
*Anthriscus*	ar		1					1			
*Apium*	ar				1					1	
*Arum*	nb				1					1	1
*Asparagus*	b		1		1		1				
*Asphodelus*	nb					1					
*Atriplex*	nb					1	1				
*Beta*	nb					1					
*Brassica*	b					1	1				
*Capparis*	b						1			1	
*Capsella*	b										1
*Centaurea*	b			1		1					1
*Chaerophyllum*	nb							1			
*Chenopodium*	nb			1				1			
*Chondrilla*	b			1							
*Cichorium*	b		1	1	1		1		1	1	
*Cirsium*	b				1						
*Crepis*	b	1					1				
*Cyclamen*	nb				1				1		
*Cynara*	b		1			1				1	
*Daucus*	ar		1								
*Diplotaxis*	b					1	1				
*Eremurus*	nb										1
*Eruca*	b			1					1		
*Eryngium*	b			1	1						
*Falcaria*	ar							1			
*Foeniculum*	ar	1	1	1	1	1	1				
*Glebionis*	b		1								
*Gundelia*	b			1	1				1		1
*Helminthotheca*	b			1							
*Heracleum*	ar							1			
*Hirschfeldia*	b		1				1				
*Hyoseris*	nb						1				
*Hypochoeris*	b	1					1				
*Lactuca*	b		1	1					1	1	
*Lapsana*	nb	1									
*Lathyrus*	nb								1		
*Launea*	b					1					
*Leontodon*	b	1		1			1				
*Leopoldia*	b	1	1								
*Lepidium*	b			1	1						
*Malva*	nb				1	1		1		1	1
*Mentha*	ar							1	1	1	1
*Micromeria*	ar				1						
*Notobasis*	b			1					1		
*Oenanthe*	ar		1								
*Onopordum*	b					1	1				1
*Origanum*	ar	1		1	1						
*Ornithogalum*	nb										1
*Papaver*	nb	1	1			1				1	1
*Plantago*	nb			1							1
*Polygonum*	nb										1
*Portulaca*	nb	1	1	1							
*Prasium*	ar		1								
*Pseudospermum*	b			1							
*Raphanus*	b	1		1			1				
*Reichardia*	nb	1	1				1				
*Rhagadiolus*	b			1							
*Rhamphospermum* ?	nb			1							
*Rheum*	nb										1
*Rorippa*	b			1	1					1	1
*Rumex*	nb		1	1	1	1				1	1
*Ruscus*	b						1				
*Salvia*	ar								1		
*Scandix*	ar	1	1		1						
*Scolymus*	b			1			1			1	
*Scorzonera*	nb			1		1					
*Silene*	nb						1				
*Silybum*	b			1		1	1				1
*Sinapis*	b	1	1	1					1	1	
*Solanum*	b	1	1								
*Sonchus*	b	1	1			1	1				
*Taraxacum*	b			1							
*Thymus*	ar				1						1
*Tordylium*	ar	1	1								
*Trifolium*	nb							1			1
*Tragopogon*	nb			1				1			1
*Urospermum*	b			1			1				
*Urtica*	nb							1	1		1
*Veronica*	nb									1	
*Vicia*	nb								1		1

ar: aromatic; b: bitter; nb: non-bitter; 1: recorded traditional/local food use; empty cells: no local food use recorded according to the analyzed literature (see Methods); ?: doubtful entry and/or misidentification (*Rhamphospermum arvense* Andrz. ex Besser is a synonym for *Sinapis arvensis* L., but Lebanese authors attributed this taxon to Boraginaceae and a different genus could be meant); the study site letter codes correspond to those in Table 1 and Figure 4.

## Data Availability

All the data are provided in the article.
